# *SCN1A*-Related Epilepsy: Novel Mutations and Rare Phenotypes

**DOI:** 10.3389/fnmol.2022.826183

**Published:** 2022-05-19

**Authors:** Rui Ma, Yiran Duan, Liping Zhang, Xiaohong Qi, Lu Zhang, Sipei Pan, Lehong Gao, Chaodong Wang, Yuping Wang

**Affiliations:** ^1^Department of Neurology, Xuanwu Hospital, Capital Medical University, Beijing, China; ^2^Beijing Key Laboratory of Neuromodulation, Beijing, China; ^3^Department of Pediatrics, Xuanwu Hospital, Capital Medical University, Beijing, China

**Keywords:** epilepsy, *SCN1A* gene, novel mutation, BECTS, cohort

## Abstract

**Objectives:**

To expand the genotypes and phenotypes of sodium voltage-gated channel alpha subunit 1 (SCN1A)-related epilepsy.

**Methods:**

We retrospectively collected the clinical and genetic information of 22 epilepsy patients (10 males, 12 females; mean: 9.2 ± 3.9 years; 3.9–20.3 years) carrying 22 variants of SCN1A. SCN1A mutations were identified by next-generation sequencing.

**Results:**

Twenty-two variants were identified, among which 12 have not yet been reported. The median age at seizure onset was 6 months. Sixteen patients were diagnosed with Dravet syndrome (DS), two with genetic epilepsy with febrile seizures plus [one evolved into benign epilepsy with centrotemporal spikes (BECTS)], one with focal epilepsy, one with atypical childhood epilepsy with centrotemporal spikes (ABECTS) and two with unclassified epilepsy. Fourteen patients showed a global developmental delay/intellectual disability (GDD/ID). Slow background activities were observed in one patient and epileptiform discharges were observed in 11 patients during the interictal phase.

**Significance:**

This study enriches the genotypes and phenotypes of SCN1A-related epilepsy. The clinical characteristics of patients with 12 previously unreported variants were described.

## Introduction

*SCN1A* is a member of the voltage-gated sodium channel (VGSC) gene family (OMIM:182389) and has been mapped to 2q24.3. *SCN1A* is the most clinically relevant gene in a wide spectrum of epilepsy phenotypes ranging from febrile seizures to Dravet syndrome (DS) (Aljaafari et al., 2017). Baulac et al. (1999) and Moulard et al. (1999) reported 2 unrelated families with generalized epilepsy with febrile seizures plus those who showed linkage to a locus on chromosome 2q21-q33. Escayg et al. (2000) identified 2 missense mutations in the *SCN1A* gene of these two families in 2000, marking *SCN1A* as a new disease gene for human inherited epilepsy. Since then, a wide variety of mutations of *SCN1A* from epilepsy patients have been identified. More than 80% of patients with DS have pathogenic variants (or mutations) in *SCN1A* (Scheffer and Nabbout, 2019). Data from a cohort of 363 Chinese DS patients in 2015 showed that 70.3% of the patients carried potentially pathogenic mutations in *SCN1A*, with a total of 223 mutations (Xu et al., 2015). As of 2015, 1727 *SCN1A* mutations had been identified in epilepsy patients. Patients with mild genotypes have a high frequency of missense mutations, which do not result in protein truncation. For more severe phenotypes, missense mutations occur less frequently. In addition, missense mutations are found in severe phenotypes, such as DS, with a higher potential to occur in the pore region of Nav1.1 than those occurring in mild phenotypes (Meng et al., 2015). However, the genotypes and phenotypes of *SCN1A* have not been completely identified. In this study, we elaborate on the clinical manifestations of 22 mutations of *SCN1A* in 22 patients in a Chinese cohort and provide more novel genotypes and phenotypes of *SCN1A*-related epilepsy.

## Materials and Methods

### Participants

Patients with epilepsy with *SCN1A* heterozygous variants were enrolled at the Neurology and Pediatric Department of Xuanwu Hospital Capital Medical University between September 2015 and November 2018. In the cohort, 367 patients with epilepsy without acquired factors were assessed by the epilepsy panel. Of these, 22 patients carried *SCN1A* variants. Clinical registrations, including name, sex, date of birth, perinatal conditions, age at the onset of seizures, clinical manifestations, family history, genetic data, video electroencephalography (EEG), magnetoencephalography (MEG), brain magnetic resonance imaging (MRI), and therapeutic regimens, were established for all patients without acquired factors. Follow-up clinical information was collected online or by telephone call.

### Genetic Analysis

*SCN1A* mutation screening was performed using next-generation sequencing of epilepsy-associated genes or whole-exome sequencing. Variants were validated using Sanger sequencing. The *SCN1A* isoform was referenced (NM_001202435 and GRCh37/hg19). All the samples were sequenced on an Illumina Nova series platform (Illumina, San Diego, CA, United States) by Kangso (Beijing, China). We analyzed the data as follows. Synonymous changes and single nucleotide polymorphisms with a minor allele frequency greater than 5% were removed.^1^ The clinical significance of the identified variants was interpreted according to the guidelines set out by the American College of Medical Genetics. The pathogenicity of the identified variants was predicted using the Mutation Taster server,^2^ Polymorphism Phenotyping version 2 (Polyphen-2),^3^ PROVEAN, and Sorting Intolerant From Tolerant (SIFT).^4^ *SCN1A* variants identified in the patients were compared with those identified in a comparison group of approximately 150,000 individuals from the Genome Aggregation Database and *SCN1A* mutation database.^5^

### Ethical Issues

This research was approved by the Ethics Committee of Xuanwu Hospital Capital Medical University. Written informed consent was obtained from the parents or guardians of all patients included in this study.

## Results

### Clinical Features

The 22 patients (10 males, 12 females; mean: 9.2 ± 3.9 years; 3.9–20.3 years) were from 22 unrelated families. Demographic and clinical characteristics are summarized in Supplementary Table 1. The median age at seizure onset and sampling was 6 months and 9 years, respectively. The first seizure type varied among the patients with an *SCN1A* mutation: febrile seizures in sixteen patients, myoclonic seizure in one patient, simple partial seizure in one patient and secondary generalized tonic-clonic seizure (GTCS) in one patient. Status epilepticus was present in 13 patients. For seizure-precipitating factors, low-grade fever in ten patients, vaccines in two patients and hot baths in twelve patients were identified.

All 22 patients had normal perinatal period and 11 patients had a family history of febrile seizures or epilepsy. The father and brother of patient 27 shared the same *SCN1A* variant with the proband with a history of febrile seizures in childhood. The twin sister of patient 28 with DS carrying the same *SCN1A* (p.Arg1892Ter) heterozygous mutation died from a sudden unexpected death in epilepsy (SUDEP) at the age of 16. The father of patient 22 suffered febrile seizures plus and became seizure-free at 8 years of age.

No patient had a developmental delay before seizure onset. Fourteen patients showed global developmental delay/intellectual disability (GDD/ID) during the disease course. These patients showed a delay in at least two of the following domains: motor skills, speech and language, cognitive skills, and social and emotional skills (Moeschler and Shevell, 2014). Patient 5 had autistic features in addition to their intellectual disability and received special education.

### Video Electroencephalography and Brain Imaging

**FIGURE 1:**
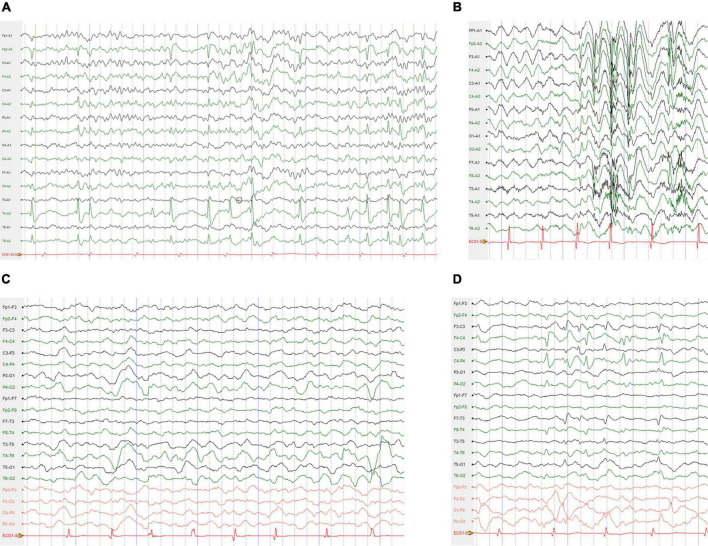
Typical electroencephalogram (EEG) changes in the cases with *SCN1A* mutations. **(A)** The interictal EEG for the patient 29 with BECTS obtained at the age of 9 years showed right mid-temporal spikes during sleep. **(B)** Interictal EEG for the patient 7 with DS obtained at the age of 5 years showed high-voltage generalized 3–4 Hz polyspike-and-waves. **(C)** Interictal EEG for the patient 12 with DS obtained at the age of 4 years showed bilateral occipital and posterior temporal 2–3.5 Hz slow waves. **(D)** Interictal EEG for the patient 6 with ABECTS obtained at the age of 8 years showed independently bilateral central and mid-temporal spikes.

Electroencephalography was obtained in 17 patients, and abnormalities were detected in 12 patients. Slow background activity was observed except for epileptiform discharges in one patient. All 11 patients had epileptiform discharges during the interictal phase. Generalized spike waves, polyspike-and-waves were captured in one patient. Focal or multifocal epileptic discharges were present in six patients. Both focal and generalized epileptic discharges were observed in four patients. Clinical seizures were captured in four patients. Two patients (9 and 25) exhibited focal to bilateral tonic-clonic seizures. Patient 1 experienced myoclonic seizures. Patient 7 had myoclonic seizures, eyelid myoclonic seizures and automatism seizures at different times. Typical electroencephalogram changes in four cases with *SCN1A* mutations are shown in Figure 1.

Among the 22 patients with brain MRI results, 17 showed a normal MRI. The MRI abnormalities included small left occipital gyrus (patient 21), left hippocampus higher signal in FLAIR (patient 18), post-operative changes of bilateral frontal and parietal lobe and corpus callosotomy (patient 8), slightly small bilateral hippocampus (patient 7) and abnormal signal in posterior horn of bilateral ventricles (patient 4).

### Phenotypic Spectrum

The phenotypic spectrum of patients with *SCN1A* variants included sixteen (72.7%) with DS, two (9.1%) with genetic epilepsy with febrile seizures plus [one evolved into benign epilepsy with centrotemporal spikes (BECTS)], one (4.5%) with focal epilepsy, one (4.5%) with atypical childhood epilepsy with centrotemporal spikes (ABECTS) and two (9.1%) with unclassified epilepsy.

### Genetic Analysis

**FIGURE 2:**
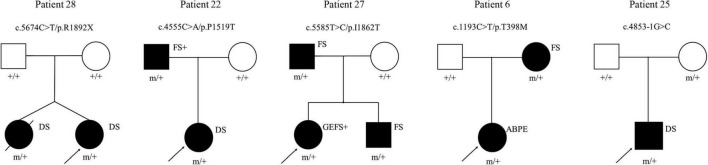
Pedigrees of the cases with inherited *SCN1A* variants and their corresponding phenotypes. ABPE, atypical benign partial epilepsy; DS, dravet syndrome; FS, febrile seizures; FS+, febrile seizures plus; GEFS+: generalized epilepsy with febrile seizures plus. □ represents female; □ represents male; 🌑 and ■ represents affected individuals; arrow represents proband; m/+ represents heterozygous mutation, +/+ represents wild type.

**FIGURE 3:**
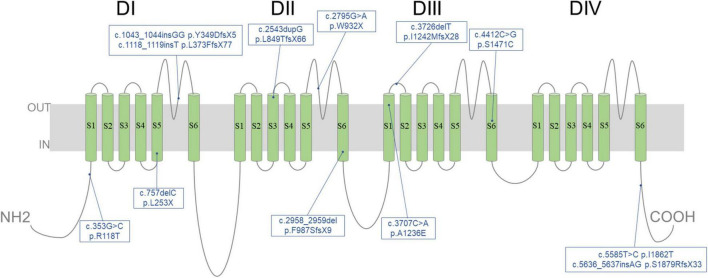
Location of 12 novel variants identified in *SCN1A* in our cohort. Schematic diagram illustrating the transmembrane topology of a voltage-gated sodium channel and location of novel variants characterized in this study.

All patients underwent genetic sequencing and carried *SCN1A* heterozygous mutations. Twenty-two kinds of pathogenic mutations were identified in the *SCN1A* mutations, including eleven missense, four non-sense, six frameshift and one splicing site mutation. Seventeen were confirmed to be *de novo*, four were inherited (one from his unaffected mother, three from their affected parents with febrile seizures or febrile seizures plus) (Figure 2) and one was unknown. The twelve novel variants (location of novel variants shown in Figure 3) and ten previously reported *SCN1A* variants are summarized in Table 1 and Supplementary Table 1. Twenty-one identified variants were likely to cause changes in the Nav1.1 protein, eight of which were in the pore region (reentrant loop between segment 5 and segment 6, and segment 6), one in the voltage sensory (segment 4), six in the transmembrane segments, two in the linker regions of domains, three in the C-terminal domain and one in the N-terminal domain.

**TABLE 1:** Summary of 12 unreported *SCN1A* variants.

Patient number	cDNA	Protein	Type of mutation	Inheritance	ACMG-based classification	SIFT;Polyphen;Mutation taster
4	1043_1044insGG	Tyr349AspfsTer5	Frameshift	*De novo*	PAT	−; −; −
5	1118_1119insT	Leu373PhefsTer77	Frameshift	*De novo*	PAT	−; −; −
8	757delC	Leu253Ter	Non-sense	*De novo*	PAT	−; −; −
9	2543dupG	Leu849ThrfsTer66	Frameshift	*De novo*	PAT	−; −; −
11	2795G > A	Trp932Ter	Non-sense	*De novo*	PAT	−; −; −
13	2958_2959del	Phe987SerfsTer9	Frameshift	*De novo*	PAT	−; −; −
16	353G > C	Arg118Thr	Missense	*De novo*	LP	Deleterious; Probably damaging; Disease causing
18	3707C > A	Ala1236Glu	Missense	*De novo*	PAT	Damaging; Probably damaging; Disease causing
19	3726delT	Ile1242MetfsTer28	Frameshift	*De novo*	PAT	−; −; −
21	4412C > G	Ser1471Cys	Missense	*De novo*	PAT	Deleterious; Probably damaging; Disease causing
27	5585T > C	Ile1862Thr	Missense	Paternal	LP	Damaging; Probably damaging; Disease causing
29	5636_5637insAG	Ser1879ArgfsTer33	Frameshift	*De novo*	PAT	−; −; −

*ACMG, American College of Medical Genetics and Genomics; LP, likely pathogenic; PAT, pathogenic.*

Variants of uncertain significance of *SCN1B* (c.566C > T, p.Thr189Met) and *SCN9A* (c.5678G > A, p.Arg1893His) were also detected in two patients (patients 4 and 16) and one patient (patient 10), respectively, all with DS.

### Correlation Between Genotype and Phenotype

In our cohort, eleven patients carried *SCN1A* mutations (non-sense, frameshift and splicing mutation), which might cause more severe protein structural changes. Eight patients (72.7%) were diagnosed with DS, one (9.1%) with generalized epilepsy with febrile seizures plus (GEFS +) converting to BECTS and two (18.2%) with unclassified epilepsy. Three (75%) of four patients with missense mutations in the pore region of the Nav1.1 channel had DS and one (25%) had ABECTS. Five (71.4%) of seven patients with missense mutations in other regions had DS, one (14.3%) had focal epilepsy and one (14.3%) had GEFS +.

### Treatment and Follow-Up

The age at which the final follow-up was taken of the 19 patients in our cohort ranged from 3 to 19 years. The mean follow-up period was 43 months. Eight patients were seizure-free for 5 months to 3 years. Patients 1 with DS had self-remission without antiepileptic medication. Seven patients were seizure-free with antiepileptic medication: two patients with DS (patients 12 and 18) and patient 29 with BECTS had valproate and levetiracetam combination therapy; patients 27 with GEFS + had levetiracetam monotherapy; two patients with DS (patients 14 and 25) were on valproate and topiramate or valproate, levetiracetam and clobazam therapy; and one patient with unclassified epilepsy accompanied by right limb dysplasia and right external auditory canal atresia (patient 13) had valproate, levetiracetam and clobazam therapy.

Eleven patients still had seizures, and their age at last follow-up ranged from 3 to 19 year 5 mo. Eight of these patients had tried at least three antiepileptic drugs. Two patients (patients 16 and 22) with DS presented increased myoclonic seizures after exposure to oxcarbazepine. Patient 8 with DS underwent epileptic lobectomy (pathological result suggested focal cortical dysplasia type I), corpus callosum resection, vagus nerve stimulation and acupuncture. She still experienced weekly GTCSs.

## Discussion

The spectra of phenotypes and genotypes of *SCN1A* mutations have been expanding. As Meng et al. (2015) counted in 2015, 1727 *SCN1A* mutations have been identified in epilepsy patients. In this study, 12 unreported mutations from 12 patients were identified, and the clinical features and mutations of the patients were described. BECTS and ABECTS are both parts of the spectrum of idiopathic rolandic epilepsy syndromes (IRES) (Gobbi et al., 2006), which are related to several genes, such as recombinant ionotropic glutamate receptor, *N*-methyl-D-aspartate 2A (*GRIN2A*), γ-aminobutyric acid A receptor (*GABAA-R*), DEP domain-containing 5 (*DEPDC5*), and RNA binding protein fox-1 homolog 1/3 (*RBFOX1/3*) (Lal et al., 2013, 2014; Lemke et al., 2013; Reinthaler et al., 2015). One patient from a GEFS + family carried with a pathogenic heterozygous *SCN1A* (c.2624C > A) variant was diagnosed with ABECTS (Kivity et al., 2017). Patients with *SCN1A* (p.R604H, p.T1250M and p.T1174S) variants were reported to have Rolandic epilepsy (Lal et al., 2016). IRES is a rare phenotype of *SCN1A* variants compared to DS. Two patients in our cohort who presented with BECTS and ABECTS carried the *SCN1A* variants (c.5636_5637insAG, p.Ser1879ArgfsX33; c.1193C > T, p.Thr398Met), respectively, adding strong evidence that the *SCN1A* variants might be responsible for IRES.

To determine genotype-phenotype associations in *SCN1A*-related epilepsy, some investigators have attempted to make a prognosis based on *SCN1A* mutations. For instance, Cetica et al. (2017) reported that truncating mutations result in earlier onset disease and a significantly higher risk of developing DS. A study by Meng et al. (2015) indicated that missense mutations in voltage sensory and ion-pore regions are associated with the DS phenotype rather than GEFS +. The frequency of missense *SCN1A* mutations in the pore region of the Nav1.1 channel in DS patients was 54.1% (Meng et al., 2015). In our cohort, most patients with truncating mutations and missense mutations in pore regions presented with a more severe DS phenotype, which corresponded with previous reports. The mutation in patient 25 was inherited from his mother without epilepsy-related phenotype but with migraine, indicating the case of phenotypic heterogeneity of this gene mutation. A recent systematic review (Hasırcı Bayır et al., 2021) summarized six families of 33 patients with mutations in the *SCN1A* gene related to epilepsy and familial hemiplegic migraine (FHM). Recent works showed the role of hyperactivity of GABAergic interneurons in a mechanism of cortical spreading depression (CSD) initiation, which is relevant as a pathological mechanism of SCN1A mutations (Jansen et al., 2020; Chever et al., 2021). The pathogenesis of the mutations identified in our study remains to be further investigated.

The VGSC subtype Nav1.7 is encoded by *SCN9A*, which is well known to be involved in the generation, development, and maintenance of pain responses (Bang et al., 2018; Chang et al., 2018). *SCN9A* was proven to be both a cause of febrile seizure and variable epilepsy phenotypes and a partner with *SCN1A* in DS (Singh et al., 2009; Yang et al., 2018). *SCN1B* encodes the VGSCβ1 and β1B non-pore-forming subunits. Early infantile developmental and epileptic encephalopathy resulting from homozygous *SCN1B* loss-of-function variants has a more severe clinical phenotype with earlier onset than typical DS (Aeby et al., 2019). The *SCN1B* (p.Thr189Met) variant was detected in sudden unexplained nocturnal death syndrome (Liu et al., 2014) and atrial fibrillation cases (Hayashi et al., 2015). This gain-of-function variant was predicted to lower the threshold potential for cellular excitability (Hayashi et al., 2015). Three patients carried mutations in not only *SCN1A* but also *SCN1B* and *SCN9A*. Whether mutations in *SCN1B* and *SCN9A* influence the phenotype cannot be defined unless functional studies are performed. Further investigations of these mutations should be conducted in the future as one of our future research directions.

Sudden unexpected death in epilepsy has been reported to account for approximately 2–18% of all epilepsy-related deaths (Gaitatzis and Sander, 2004) and has a higher incidence in DS (Dravet et al., 2005). SUDEP in GEFS + (Hindocha et al., 2008) and DS (Le Gal et al., 2010) patients with *SCN1A* mutations have also been reported. SUDEP was not observed in patients in our cohort but in a twin sister of patient 28 with DS carrying the same *SCN1A* heterozygous mutation at the age of 16. The specific reason for death in our patient’s twin sister is unknown. The proposed mechanisms of SUDEP include (Tomson et al., 2008): effects of long-standing seizure disorder; predisposition to SUDEP, incidental or related to etiology of epilepsy; factors related to drug treatment; unknown factors that transform a seizure into a fatal event; precipitating seizure. Ultimately, apnea/hypoxia and cardiac arrhythmia with electrocerebral shutdown cause SUDEP. Patients with DS seem predisposed to SUDEP, with an imbalance of cardiac autonomic function with decreased heart rate variability and increased P wave and QT dispersion compared with other forms of epilepsy (Delogu et al., 2011; Ergul et al., 2013).

Our cohort was not large, and a significant pattern between phenotype severity and mutation location may not be concluded. Large-scale prospective studies are needed to assess the effect of treatment on development in the long term.

This study involved a cohort of 22 patients carrying *SCN1A* variants from a single center. We described the details of the clinical and genetic alterations of patients with some atypical symptoms, mainly BECTS and ABECTS. We also identified 12 novel variants of *SCN1A* in a Chinese population, extending the phenotypic and genotypic spectra. In addition, we reported the prognosis of the 19 patients with a mean follow-up period of 43 months. One case of SUDEP with variants of p.Arg1892Ter was described, reminding us that clinical methods to predict SUDEP risks need to be developed. Supervision in appropriate cases and provision of balanced information to patients and relatives are also of vital importance.

## Data Availability Statement

The datasets presented in this study can be found in online repositories. The names of the repository/repositories and accession number(s) can be found below: GenBank, OM280336–OM280357.

## Ethics Statement

The studies involving human participants were reviewed and approved by the Ethics Committee of Xuanwu Hospital Capital Medical University. Written informed consent to participate in this study was provided by the participants’ legal guardian/next of kin. Written informed consent was obtained from the individual(s), and minor(s)’ legal guardian/next of kin, for the publication of any potentially identifiable images or data included in this article.

## Author Contributions

RM: writing – original draft and visualization. YD, LiZ, XQ, LuZ, and SP: resources. LG: writing – reviewing and editing and validation. CW: data curation and conceptualization. YW: supervision, project administration, and funding acquisition. All authors contributed to the article and approved the submitted version.

## Conflict of Interest

The authors declare that the research was conducted in the absence of any commercial or financial relationships that could be construed as a potential conflict of interest.

## Publisher’s Note

All claims expressed in this article are solely those of the authors and do not necessarily represent those of their affiliated organizations, or those of the publisher, the editors and the reviewers. Any product that may be evaluated in this article, or claim that may be made by its manufacturer, is not guaranteed or endorsed by the publisher.

## Footnotes

1
http://www.ncbi.nlm.nih.gov/projects/SNP

2
http://www.mutationtaster.org/

3
http://genetics.bwh.harvard.edu/pph2/

4
http://sift.jcvi.org/

5
http://scn1a.caae.org.cn/
