# Supplementary material for: SCN1A-Related Epilepsy: Novel Mutations and Rare Phenotypes
Source: Front Mol Neurosci. 2022 May 19;15:826183. doi: 10.3389/fnmol.2022.826183 (PMC9162153; doi:10.3389/fnmol.2022.826183)
Supplement: Supplementary file 1 [file Table_1.docx]

**Table S1:** Summary of 10 reported *SCN1A* mutations.

| Patient number | cDNA | Protein | Type of mutation | Inheritance | Previous report | ACMG-based classification | SIFT；Polyphen；Mutation taster |
| --- | --- | --- | --- | --- | --- | --- | --- |
| 1 | c.568T>C | p.Trp190Arg | Missense | De novo | DS^1-3^ | PAT | Damaging; Probably damaging; disease causing |
| 6 | c.1193C>T | p.Thr398Met | Missense | Maternal | IE^4^ | LP | Damaging; Probably damaging; disease causing |
| 7 | c.1277A>G | p.Tyr426Cys | Missense | De novo | DS^2^  Epilepsy^5^ | PAT | Damaging; Probably damaging; disease causing |
| 10 | c.2584C>T | p.Arg862Ter | Nonsense | De novo | GEFS+^7^  DS^2, 8, 9^ | PAT | -; -; - |
| 12 | c.2836C>T | p.Arg946Cys | Missense | De novo | DS^2, 3, 8-11^  Epilepsy^5^ | PAT | Deleterious; Probably damaging; disease causing |
| 14 | c.2959T>C | p.Phe987Leu | Missense | De novo | DS^2^ | PAT | Deleterious; Probably damaging; disease causing |
| 22 | c.4555C>A | p.Pro1519Thr | Missense | Paternal | DS^13^  Epilepsy^5^ | LP | Damaging; Probably damaging; disease causing |
| 24 | c.4762T>C | p.Cys1588Arg | Missense | NA | DS^10, 14^  EE^15^  Epilepsy^5^ | PAT | Deleterious; Probably damaging; disease causing |
| 25 | c.4853-1G>C | - | Splicing | Maternal | DS^16^  Epilepsy^2^ | PAT | ‘-; -; - |

ACMG: American College of Medical Genetics and Genomics; DS: Dravet syndrome; EE: epileptic encephalopathy; GEFS+: generalized epilepsy with febrile seizures plus; IE: intractable epilepsy; LP: likely pathogenic; NA: not available; PAT: pathogenic.

**Reference**

1. Xu XJ, Zhang YH, Sun HH, Liu XY, Jiang YW, Wu XR. Genetic and phenotypic characteristics of SCN1A mutations in Dravet syndrome. Zhonghua yi xue yi chuan xue za zhi = Zhonghua yixue yichuanxue zazhi = Chinese journal of medical genetics. 2012;29(6):625-30.

2. Wang JW, Shi XY, Kurahashi H, Hwang SK, Ishii A, Higurashi N, et al. Prevalence of SCN1A mutations in children with suspected Dravet syndrome and intractable childhood epilepsy. Epilepsy research. 2012;102(3):195-200.

3. Fukuma G, Oguni H, Shirasaka Y, Watanabe K, Miyajima T, Yasumoto S, et al. Mutations of neuronal voltage-gated Na+ channel alpha 1 subunit gene SCN1A in core severe myoclonic epilepsy in infancy (SMEI) and in borderline SMEI (SMEB). Epilepsia. 2004;45(2):140-8.

4. Kang KW, Kim W, Cho YW, Lee SK, Jung KY, Shin W, et al. Genetic characteristics of non-familial epilepsy. PeerJ. 2019;7:e8278.

5. Lindy AS, Stosser MB, Butler E, Downtain-Pickersgill C, Shanmugham A, Retterer K, et al. Diagnostic outcomes for genetic testing of 70 genes in 8565 patients with epilepsy and neurodevelopmental disorders. Epilepsia. 2018;59(5):1062-71.

6. Kwong AK, Fung CW, Chan SY, Wong VC. Identification of SCN1A and PCDH19 mutations in Chinese children with Dravet syndrome. PloS one. 2012;7(7):e41802.

7. Orrico A, Galli L, Grosso S, Buoni S, Pianigiani R, Balestri P, et al. Mutational analysis of the SCN1A, SCN1B and GABRG2 genes in 150 Italian patients with idiopathic childhood epilepsies. Clinical genetics. 2009;75(6):579-81.

8. Lee HF, Chi CS, Tsai CR, Chen CH, Wang CC. Electroencephalographic features of patients with SCN1A-positive Dravet syndrome. Brain & development. 2015;37(6):599-611.

9. Xu X, Zhang Y, Sun H, Liu X, Yang X, Xiong H, et al. Early clinical features and diagnosis of Dravet syndrome in 138 Chinese patients with SCN1A mutations. Brain & development. 2014;36(8):676-81.

10. Zuberi SM, Brunklaus A, Birch R, Reavey E, Duncan J, Forbes GH. Genotype-phenotype associations in SCN1A-related epilepsies. Neurology. 2011;76(7):594-600.

11. Volkers L, Kahlig KM, Verbeek NE, Das JH, van Kempen MJ, Stroink H, et al. Nav 1.1 dysfunction in genetic epilepsy with febrile seizures-plus or Dravet syndrome. Eur J Neurosci. 2011;34(8):1268-75.

12. Holland KD, Bouley TM, Horn PS. Comparison and optimization of in silico algorithms for predicting the pathogenicity of sodium channel variants in epilepsy. Epilepsia. 2017;58(7):1190-8.

13. Moehring J, von Spiczak S, Moeller F, Helbig I, Wolff S, Jansen O, et al. Variability of EEG-fMRI findings in patients with SCN1A-positive Dravet syndrome. Epilepsia. 2013;54(5):918-26.

14. Marini C, Mei D, Temudo T, Ferrari AR, Buti D, Dravet C, et al. Idiopathic epilepsies with seizures precipitated by fever and SCN1A abnormalities. Epilepsia. 2007;48(9):1678-85.

15. Mercimek-Mahmutoglu S, Patel J, Cordeiro D, Hewson S, Callen D, Donner EJ, et al. Diagnostic yield of genetic testing in epileptic encephalopathy in childhood. Epilepsia. 2015;56(5):707-16.

16. Mancardi MM, Striano P, Gennaro E, Madia F, Paravidino R, Scapolan S, et al. Familial occurrence of febrile seizures and epilepsy in severe myoclonic epilepsy of infancy (SMEI) patients with SCN1A mutations. Epilepsia. 2006;47(10):1629-35.

17. Nicita F, Spalice A, Papetti L, Ursitti F, Parisi P, Gennaro E, et al. Genotype-phenotype correlations in a group of 15 SCN1A-mutated Italian patients with GEFS+ spectrum (seizures plus, classical and borderline severe myoclonic epilepsy of infancy). Journal of child neurology. 2010;25(11):1369-76.

18. Sakakibara T, Nakagawa E, Saito Y, Sakuma H, Komaki H, Sugai K, et al. Hemiconvulsion-hemiplegia syndrome in a patient with severe myoclonic epilepsy in infancy. Epilepsia. 2009;50(9):2158-62.

19. Harkin LA, McMahon JM, Iona X, Dibbens L, Pelekanos JT, Zuberi SM, et al. The spectrum of SCN1A-related infantile epileptic encephalopathies. Brain : a journal of neurology. 2007;130(Pt 3):843-52.

20. Sugawara T, Mazaki-Miyazaki E, Fukushima K, Shimomura J, Fujiwara T, Hamano S, et al. Frequent mutations of SCN1A in severe myoclonic epilepsy in infancy. Neurology. 2002;58(7):1122-4.
